# Chinese expert consensus on the treatment of immune‑mediated inflammatory diseases with Janus kinase inhibitors

**DOI:** 10.1515/rir-2026-0028

**Published:** 2026-10-22

**Authors:** Xiaofeng Zeng, Jianzhong Zhang, Minhu Chen, National Clinical Research Center for Rheumatic and Autoimmune Diseases (Peking Union Medical College Hospital), Chinese Association of Rheumatology and Immunology PhysiciansDermatology Professional Committee of Chinese Rehabilitation Medical Association

**Affiliations:** Peking Union Medical College Hospital, Peking Union Medical College, Chinese Academy of Medical Sciences, Beijing, China; Department of Dermatology, Peking University People′s Hospital, Beijing, China; Department of Gastroenterology, the First Affiliated Hospital, Sun Yat‑sen University, Guangzhou, Guangdong Province, China

**Keywords:** Janus kinase inhibitors, immune-mediated inflammatory diseases, treatment, expert consensus

## Abstract

In recent years, Janus kinase (JAK) inhibitors have been approved for the treatment of various immune-mediated inflammatory diseases (IMIDs), including rheumatoid arthritis, axial spondyloarthritis, psoriatic arthritis, juvenile idiopathic arthritis, giant cell arteritis, atopic dermatitis, alopecia areata, psoriasis, and inflammatory bowel disease. To standardize the clinical application of JAK inhibitors for IMIDs in China, this consensus group has developed the “Chinese Expert Consensus on the Treatment of Immune-Mediated Inflammatory Diseases with Janus Kinase Inhibitors.” This consensus is formulated based on relevant evidence-based medicine, the latest research data from both domestic and international studies, and the clinical experience of the expert panel. It covers key aspects such as treatment protocols, safety management, and medication use in special patient populations. The aim of this consensus is to assist physicians in making rational clinical decisions regarding the use of JAK inhibitors.

Immune-mediated inflammatory diseases (IMIDs) are a group of heterogeneous diseases, mainly including rheumatoid arthritis (RA), axial spondyloarthritis (axSpA), psoriatic arthritis (PsA), juvenile idiopathic arthritis (JIA), giant cell arteritis (GCA), systemic lupus erythematosus (SLE), systemic sclerosis (SSc), atopic dermatitis (AD), alopecia areata (AA), psoriasis (PsO), Crohn’s disease (CD), and ulcerative colitis (UC).^[1]^ IMIDs are caused by immune homeostasis disturbances induced by environmental factors in genetically susceptible individuals, sharing a common core pathogenesis.^[2,3]^ Specifically, the most characteristic presentations are are infiltration of immune cells in the target organs in pathology, accompanied by persistent secretion of pro-inflammatory cytokines such as tumor necrosis factor (TNF), interleukin (IL), interferon (IFN), and abnormal activation of chemokine networks,^[1,4]^ ultimately leading to chronic inflammatory responses and progressive tissue damage.

The Janus kinase (JAK), signal transducer and activator of transcription (STAT) pathway regulate the gene transcription of key cytokines and chemokines, playing an important role in the occurrence and development of IMIDs.^[5, 6, 7]^ The JAK family includes four subtypes: JAK1, JAK2, JAK3, and TYK2, each possessing distinct functions: JAK1 is involved in the signal transduction of type I and II IFNs and the γc chain receptor family-related cytokines. So inhibition of JAK1 can improve inflammation and protect joint tissues; JAK2 is involved in erythropoiesis, hematopoietic cell development, and cytokine signal transmission; JAK3 regulates γc chain receptor-related signal transduction and the development and function of T cells and NK cells; TYK2 participates in the signal transduction of type I and II IFNs as well as IL-12 and IL-23.^[8,9]^ As core molecules in the signal transduction of multiple pro-inflammatory cytokine receptors, JAK phosphorylation can be specifically inhibited by JAK inhibitors, thereby blocking the activation of signaling pathways after the binding of cytokines to receptors, achieving precise regulation of immune signals (Figure 1). Currently, JAK inhibitors have become an effective treatment for various IMIDs. The JAK inhibitors are currently marketed in China and their approved indications are listed in detaile in Table 1.

**Figure 1:**
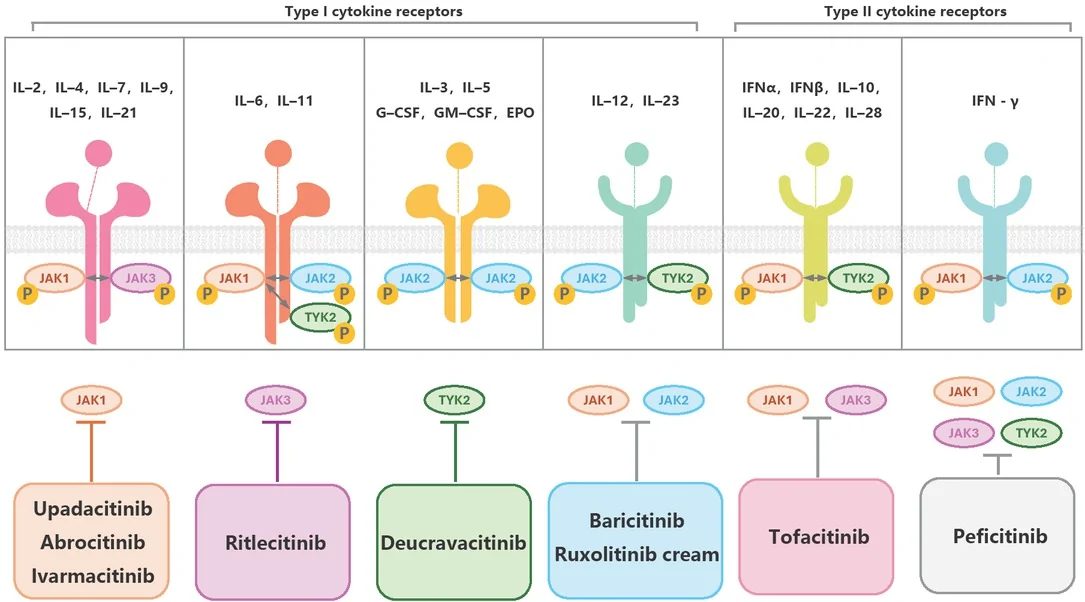
Primary targets of JAK inhibitors.^[2,6,181, 182, 183, 184, 185, 186, 187]^ JAK, Janus kinase; TYK2, Tyrosine kinase 2; IL: interleukin; IFN: interferon; G-CSF, granulocyte colony-stimulating factor; GM-CSF, granulocyte-macrophage colony-stimulating factor; EPO, erythropoietin.

**Table 1:** JAK inhibitors approved in China

Drug	Target	Indications (China)	Indications (International)	Specifications
Tofacitinib	JAK1/3	RA, PsA, AS	RA, UC, JIA, AS	5 mg, 11 mg (PR)
Baricitinib	JAK1/2	RA, AA, JIA	RA, AD, SLE, AA	2, 4 mg
Upadacitinib	JAK1	RA, PsA, AD, AS, UC, CD, nr-axSpA, GCA	RA, AD, PsA, AS, CD, UC, nr-axSpA, JIA, GCA	15, 30, 45 mg
Abrocitinib	JAK1	AD	AD	50, 100, 200 mg
Deucravacitinib	TYK2	PsO	PsO	6 mg
Ritlecitinib	JAK3	AA	AA	50 mg
Peficitinib	Pan-JAK	RA	RA	50 mg
Ivarmacitinib	JAK1	AS, RA, AD, AA	N/A	4 mg

Drugs are listed by market launch date. AA, alopecia areata; AD, atopic dermatitis; AS, ankylosing spondylitis; CD, Crohn’s disease; GCA, giant cell arteritis; JAK, Janus kinase; JIA, juvenile idiopathic arthritis; N/A, not applicable; nr-axSpA, non-radiographic axial spondyloarthritis; PR, prolonged-release; PsA, psoriatic arthritis; PsO, plaque psoriasis; RA, rheumatoid arthritis; SLE, systemic lupus erythematosus; UC, ulcerative colitis.

## Consensus Development Process

### Consensus Working Group

The consensus development working group was composed of clinical experts and methodologists in the fields of rheumatology and immunology, dermatology, and gastroenterology. All working group members completed Conflict of Interest (COI) disclosure forms and declared no conflicts of interest related to this consensus. This consensus has been registered on the International Practice Guideline Registration and Transparency Platform (Registration No. PREPARE-2025CN393).

### Evidence Retrieval

To establish the core clinical questions for JAK inhibitor therapy in IMIDs, the expert panel undertook a comprehensive process. This involved a systematic review of international and Chinese guidelines, management recommendations, consensus statements, and treatment protocols. The formulation was further informed by integrating clinical experience from China, conducting extensive expert consultations, and comparing Chinese and international drug labels and scientific literature on JAK inhibitors.

The resulting clinical questions were then structured according to the Population, Intervention, Comparison, and Outcome (PICO) framework. Following this, a detailed search strategy was developed and executed to systematically search the following databases: PubMed, Embase, the Cochrane Library, ClinicalTrials. gov, the Chinese Clinical Trial Registry, the Chinese Biomedical Literature Database (CBM), Wanfang Data, China National Knowledge Infrastructure (CNKI), and the VIP Information Network. The search, covering the period up to June 27, 2025, targeted systematic reviews, meta-analyses, and relevant clinical studies, including randomized controlled trials (RCTs), cohort studies, case-control studies, and case series.

Concurrently, the panel retrieved prescribing information from the National Medical Products Administration (NMPA) of China, the U. S. Food and Drug Administration (FDA), and the European Medicines Agency (EMA). To ensure comprehensive coverage, a supplementary search was conducted on the reference lists of relevant guidelines, recommendations, and consensus statements issued by major international and Chinese academic societies. These included the American College of Rheumatology (ACR), the European Alliance of Associations for Rheumatology (EULAR), the Assessment of SpondyloArthritis international Society (ASAS), the Group for Research and Assessment of Psoriasis and Psoriatic Arthritis (GRAPPA), the American Academy of Dermatology (AAD) /National Psoriasis Foundation (NPF), the European Academy of Dermatology and Venereology (EADV), the Japanese Dermatological Association (JDA), the American Gastroenterological Association (AGA), and the Chinese Medical Association (CMA).

### Evaluation and Grading of Evidence

The methodology expert group assessed the quality of different types of included evidence using standardized tools. For systematic reviews, meta-analyses, and network meta-analyses, the risk of bias was evaluated using A MeaSurement Tool to Assess systematic Reviews (AMSTAR 2).^[10]^ For RCTs, the Cochrane Risk of Bias (RoB) tool was employed.^[11]^ Observational studies were assessed for methodological quality using the Newcastle-Ottawa Scale (NOS).^[12]^ This entire evaluation process was conducted independently by two researchers. Any disagreements were resolved through discussion or, if necessary, consultation with a third expert to reach a consensus. The quality of evidence was then graded using the Grading of Recommendations Assessment, Development and Evaluation (GRADE) methodology (Table 2).^[13]^

**Table 2:** Definition of grades for the quality of evidence

Grade	Definition
High (A)	High confidence that the estimated effect is close to the true effect.
Moderate (B)	Moderate confidence in the effect estimate; the true effect may differ from the estimate.
Low (C)	Limited confidence in the effect estimate; the true effect may be substantially different from the estimate.
Very low (D)	Very little confidence in the effect estimate; the true effect is likely to be substantially different from the estimate.

### Formation of Recommendations

The expert panel drafted initial recommendations by considering the balance of benefits and harms, evidence quality, clinical feasibility, economic costs, and patient preferences for each clinical question. These draft recommendations were then refined through hybrid (online and offline) consensus meetings. The level of agreement on each statement was systematically evaluated by the working group. The final recommendations were formed after several rounds of iterative discussion and revision.

## Consensus and Rationale

This consensus includes the overall principles of JAK inhibitor use in IMIDs and disease-specific recommendations, as shown in Table 3.

**Table 3:** Consensus on the application of JAK inhibitors in IMIDs

No.	Consensus opinion	Evidence grade
1	JAK inhibitors may be considered for patients with IMIDs who have failed conventional therapies or biologic agents and have no contraindications. These diseases include, but are not limited to, RA, axSpA, PsA, JIA, GCA, AD, AA, PsO, UC, and CD.	A
2	JAK inhibitors may be considered for the treatment of patients with more than one IMID.	D
3	After the initiation of JAK inhibitor therapy, disease activity assessment is recommended every 1–3 months, with subsequent follow-up frequency adjusted based on the assessment results. For patients who have achieved treatment targets, disease activity assessment may be performed every 3–6 months.	B
4	For adult patients with moderate to severe active RA who have inadequate response to or intolerance of csDMARDs or TNF inhibitors, JAK inhibitors were recommended as monotherapy or in combination with csDMARDs; in patients with ongoing clinical remission for ≥ 6 months, medication reduction may be considered.	A
5	For adult patients with active axSpA who have an inadequate response to or intolerance of NSAIDs or TNF inhibitors, JAK inhibitors are recommended. For patients whose disease has been stable for ≥ 6 months, dose reduction may be considered.	A
6	JAK inhibitors are recommended for adult patients with PsA who have an inadequate response to or intolerance of one or more DMARDs; dose reduction may be considered in patients who have maintained sustained remission for ≥ 1 year.	A
7	For patients with active JIA aged 2 years and older who have an inadequate response to or intolerance of csDMARDs/bDMARDs, JAK inhibitors are recommended. For patients who achieve sustained remission for ≥ 1 year, dose reduction may be considered. It is recommended that children with polyarticular or oligoarticular JIA continue treatment for 2 years after achieving clinical remission.	A
8	JAK inhibitors are recommended for the treatment of new-onset or recurrent GCA in adults.	B
9	JAK inhibitors are recommended for the treatment of moderate-to-severe AD. Patients prioritized for JAK inhibitors include: (1) those requiring rapid relief of disease symptoms; (2) those unwilling to undergo subcutaneous injections or phototherapy; (3) those who have developed immune drift from the use of biologic agents. After achieving an adequate response following 3 to 6 months of standardized treatment, the dose can be reduced for maintenance. If discontinuation is considered, an intermittent dosing regimen, such as once every other day or twice weekly, is recommended for gradual tapering.	A
10	JAK inhibitors are recommended for the treatment of patients with moderate to severe AA. To reduce the risk of recurrence, treatment should be maintained for at least 6 to 12 months after complete hair regrowth before considering gradual dose reduction.	A
11	TYK2 inhibitors are recommended for adult patients with moderate-to-severe plaque PsO who are candidates for systemic therapy or phototherapy.	A
12	JAK inhibitors are recommended for adult patients with moderate-to-severe active UC who have an inadequate response to or are intolerant of TNF inhibitors.	A
13	JAK inhibitors are recommended for adult patients with moderate-to-severe active CD who have an inadequate response to or are intolerant of TNF inhibitors.	A
14	JAK inhibitors are conditionally recommended for the treatment of IMIDs such as hand eczema, vitiligo, SLE, dermatomyositis, and Behçet’s disease.	B
15	JAK inhibitor ointments/creams are conditionally recommended as a treatment option for patients with AA, AD, PsO, vitiligo, and hand eczema who have inadequate control with conventional therapy.	B
16	When JAK inhibitors are used in combination with biological agents or immunosuppressants, the risk of serious adverse events should be thoroughly assessed; in patients receiving concomitant glucocorticoid therapy, glucocorticoids may be considered for gradual tapering or discontinuation after achieving stable disease.	B
17	Prior to the use of JAK inhibitors, the potential risks and benefits should be adequately assessed, including thromboembolic events, severe cardiovascular events, and malignant neoplasms, and safety monitoring should be conducted. The monitoring parameters and frequency could be adjusted according to clinical needs. However, the potential differences in the types and incidence rates of adverse events among different JAK inhibitors should be noted.	B
18	Medication in special populations: (1) JAK inhibitors should be avoided in pregnant and lactating women, and medication should be discontinued at least 1 month prior to pregnancy planning (or conception); (2) elderly patients receiving JAK inhibitors should be monitored for infection, cardiovascular events, and thromboembolic risk; (3) JAK inhibitors should be used with caution in patients with severe hepatic or renal impairment; (4) live attenuated vaccines are not recommended for patients with rheumatic disorders; in patients receiving inactivated vaccines, JAK inhibitors do not need to be discontinued; (5) for patients undergoing major surgery, JAK inhibitors should be discontinued 3 days prior to surgery, and medication may be resumed after surgery if postoperative wound healing is satisfactory and there is no surgical site or systemic infection.	B

The consensus opinions are ordered according to the marketing approval date for JAK inhibitors in each disease. A, B, C, and D refer to the grades of evidence defined in Table 2. AA, alopecia areata; AD, atopic dermatitis; axSpA, axial spondyloarthritis; bDMARDs, biologic disease-modifying antirheumatic drugs; CD, Crohn’s disease; csDMARDs, conventional synthetic disease-modifying antirheumatic drugs; GCA, giant cell arteritis; IMIDs, immune-mediated inflammatory diseases; JAK, Janus kinase; JIA, juvenile idiopathic arthritis; PsA, psoriatic arthritis; PsO, psoriasis; RA, rheumatoid arthritis; TNF, tumor necrosis factor; UC, ulcerative colitis.

### Consensus 1

JAK inhibitors may be considered for patients with IMIDs who failed to respond to conventional therapies or biologic agents and have no contraindications. These diseases include, but are not limited to, RA, axSpA, PsA, JIA, GCA, AD, AA, PsO, UC, and CD (Evidence Level: A).

Although treatment strategies for IMIDs continue to evolve, multiple challenges remain. Traditional immunosuppressive agents and glucocorticoids, the cornerstones of IMIDs treatment, often show a decline in efficacy as the treatment duration extends, making it difficult to maintain long-term clinical remission or low disease activity. At the same time, these medications may exhibit potential toxicity to the hematologic, hepatic, cardiovascular, and metabolic systems, which limits their long-term use.^[14]^ With a deeper understanding of the pathogenesis of IMIDs, biologic agents—represented by TNF inhibitors, IL-23 inhibitors, and IL-17 inhibitors—have significantly changed the treatment landscape of IMIDs by specifically blocking key inflammatory cytokine signaling.^[14,15]^ However, approximately one-fifth of patients remain refractory to the currently available biological agents.^[2]^ In addition, the use of biologics may paradoxically trigger new IMIDs, driven by factors such as immune drift, with an incidence of approximately 8 per 10, 000 exposed patients.^[16]^

The therapeutic efficacy of JAK inhibitors has been well validated across various IMIDs, not only contributing to significant improvement in disease activity, but also delaying, or even arresting disease progression, providing IMID patients with novel treatment options.^[5]^ Currently, the NMPA has approved JAK inhibitors for the treatment of RA, axSpA, PsA, JIA, GCA, AD, AA, PsO, UC, CD, and other IMIDs (Table 1). In addition, in other IMID-related fields such as hand eczema, vitiligo, Sjögren’s syndrome, SLE, vasculitis, dermatomyositis, SSc, hidradenitis suppurativa, and Behçet’s disease, some clinical studies have demonstrated favorable clinical efficacy.^[17, 18, 19, 20, 21, 22, 23, 24, 25]^

### Consensus 2

JAK inhibitors may be considered for the treatment of patients with more than one IMID (Evidence Level: D).

IMIDs often share some susceptibility genes and immune pathways, leading to an increased risk of patients with one IMID developing another IMID, a phenomenon termed “polyautoimmunity”.^[26,27]^ The associations among various IMIDs were detailed in Table 4. In such patients, the diseases can affect multiple organ systems either simultaneously or sequentially. This not only results in complex and diverse clinical manifestations but also presents challenges such as conflicting drug choices and limited treatment options, thereby significantly increasing the difficulty of clinical management.^[28]^

**Table 4:** Risk of comorbidity among common immune-mediated inflammatory diseases

Patients	RA risk	AS risk	IBD risk	SLE risk	AD risk	AA risk	PsO risk	Vitiligo risk
RA	N/A	↑↑^[188]^	↑↑^[188]^	↑↑^[188, 189]^	↓^[190]^	—^[188]^	↑↑^[188]^	↑↑^[189]^
AS	R^[191]^	N/A	↑↑^[191, 192]^	R^[193]^	↔^[190]^	ND	↑^[194]^	↑↑^[196]^
IBD	↑↑^[196]^	↑↑^[196,197]^	N/A	↑^[196]^	↑↑^[190]^	↑^[198]^	↑↑^[199]^	ND
SLE	R^[200]^	R^[193]^	↔^[201,202]^	N/A	↔^[190]^	↑↑^[203]^	R^[204]^	ND
AD	↑↑^[205-207]^	ND	↑↑^[190]^	↑↑^[190]^	N/A	↑↑^[190]^	↑^[208]^	↑↑^[190]^
AA	↑^[209,210]^	—^[209]^	—^[209]^	↑↑^[211]^	↑↑^[190,209,210]^	N/A	↑^[209,210]^	↑^[209,210]^
PsO	↑↑^[212,213]^	ND	↑↑^[212,213]^	↑↑^[212,213]^	↑^[208]^	↑↑^[212,213]^	N/A	↑↑^[212,213]^
Vitiligo	↑↑^[214]^	ND	↑↑^[215,216]^	↑↑^[215]^	↑↑^[190]^	↑↑^[215]^	—^[217]^	N/A

Note: This table summarizes the risk of developing a secondary disease (column) in patients with a pre-existing baseline disease (row). Symbols indicate the nature of the risk, where ↑↑ denotes a significantly increased risk with consistent evidence; ↑ and ↓ represent a suggested increase or decrease in risk, respectively, without statistical significance; and — signifies no association. Furthermore, ↔ indicates conflicting evidence, R denotes a rare association (case reports only), ND is used when data are unavailable, and N/A marks cells as not applicable. AA, alopecia areata; AD: atopic dermatitis; AS, ankylosing spondylitis; IBD, inflammatory bowel disease; PsO, psoriasis; RA, rheumatoid arthritis; SLE, systemic lupus erythematosus.

Since JAK inhibitors block multiple cytokines signaling pathways, they have shown potential efficacy in patients with multiple IMIDs. A RCT in patients with RA secondary to Sjögren’s syndrome showed that the clinical response rate of tofacitinib was significantly superior to methotrexate (RA response rate: 97.22% *vs*. 77.78%; Sjögren’s syndrome response rate: 97.22% *vs*. 80.56%).^[29]^ However, due to the limited number of patients with multiple IMIDs, the current evidence of JAK inhibitors in this population mainly comes from case reports.

Data have shown that JAK inhibitors have demonstrated good therapeutic efficacy and safety in treating AD combined with AA,^[30, 31, 32]^ AA combined with psoriatic nail disease,^[33]^ or vitiligo,^[34]^ palmoplantar pustulosis combined with AA,^[35]^ PsO combined with AD,^[36]^ SLE combined with PsO,^[37]^ vitiligo combined with PsO,^[38]^ RA combined with focal myositis,^[39]^ and PsA combined with eczema.^[40]^ Large-scale clinical evidence is still needed in the future to support these findings.

### Consensus 3

After the initiation of JAK inhibitor therapy, it is recommended that disease activity assessment should be done every 1–3 months, the subsequent follow-up frequency should be adjusted based on the assessment results. For patients who have achieved treatment targets, disease activity assessment may be performed every 3–6 months (Evidence Level: B).

During JAK inhibitor treatment for IMIDs, dynamic monitoring of disease activity is critical for optimizing treatment strategies and ensuring therapeutic efficacy and safety. JAK inhibitors typically have a rapid onset of action, most patients would experience clinical symptom improvement within 2–4 weeks after the treatment.^[41, 42, 43]^ However, as therapeutic responses vary considerably between individuals, it is recommended to conduct disease activity assessments every 1–3 months during the initial phase of treatment to allow for timely adjustments to the regimen based on the patient’s actual response.^[44, 45, 46, 47]^ For stable patients who have achieved clinical remission or met the criteria for low disease activity, the monitoring interval may be appropriately extended to every 3–6 months to comprehensively evaluate disease control and ensure sustained remission or a low disease activity.^[44, 45, 46, 47]^

In addition, certain subsets of IMID patients require shorter monitoring intervals during the initial treatment phase. For example, as JAK inhibitors may resolve pruritus and skin lesions in AD patients more rapidly, increasing the assessment frequency in the first month (*e.g*., biweekly) can more precisely capture changes in the disease course. A previous consensus suggested that therapeutic efficacy could be assessed daily during the first week of treatment, followed by assessments at the end of weeks 2 and 4, and monthly thereafter.^[48]^ Children with active systemic JIA (sJIA) often present with multi-system involvement, such as fever, rash, and arthralgia; given the complexity and rapid progression of sJIA, close monitoring (*e.g*., weekly or every 2 weeks) is essential for the prompt identification of disease fluctuations. This allows timely treatment adjustments to prevent severe complications, such as macrophage activation syndrome (MAS).^[49,50]^

### Consensus 4

For adult patients with moderate to severe active RA who have inadequate response or intolerance to conventional synthetic disease-modifying anti-rheumatic drugs (csDMARDs) or TNF inhibitors, JAK inhibitors are recommended as monotherapy or in combination with csDMARDs. For patients with clinical remission for ≥ 6 months, medication reduction may be considered (Evidence Level: A).

The ORAL Sync trial showed that at 6 months, the ACR improvement of ≥ 20% (ACR20) response rate in patients with moderate to severe RA treated with tofacitinib 5 mg and 10 mg in combination with csDMARDs was significantly higher than that of placebo (67.4%, 70.6% *vs*. 34.1%).^[51]^ The RABUILD trial showed that at 12 weeks, the ACR20 response rate in RA patients treated with baricitinib in combination with csDMARDs was significantly higher than that of placebo (62% *vs*. 39%).^[52]^ The SELECT-NEXT trial showed that at 12 weeks, the ACR20 (64%, 66% *vs*. 36%) and the disease activity score based on 28-joint counts using C-reactive protein (DAS28-CRP) < 3.2 (48%, 48% *vs*. 17%) response rates in RA patients treated with upadacitinib 15 mg and 30 mg in combination with csDMARDs were significantly higher than that of placebo.^[53]^ Ivarmacitinib is the first highselective JAK1 inhibitor developed independently in China. A Phase III study showed that at 24 weeks, the ACR20 response rate in patients with moderate to severe RA treated with ivarmacitinib 4 mg and 8 mg in combination with csDMARDs was significantly superior to placebo (70.4%, 75.1% *vs*. 40.4%).^[54]^ In addition, JAK inhibitors could also be used as monotherapy in patients with contraindications to csDMARDs.^[55]^ A Phase III RCT study showed that at 3 months, the ACR20 response rate in RA patients treated with tofacitinib 5 mg and 10 mg as monotherapy was significantly higher than that of placebo (59.8%, 65.7% *vs*. 26.7%).^[56]^ A Phase III study showed that at 24 weeks, the ACR20 response rate in RA patients treated with baricitinib as monotherapy was significantly higher than that with methotrexate (77.0% *vs*. 62.0%).^[57]^ The RA-BALANCE trial showed that at week 12, patients with moderate-to-severe RA treated with baricitinib monotherapy showed significantly greater improvement from baseline in Patient’s Global Assessment of Disease Activity (PtGA) and patient-reported outcomes (PROs) compared to those treated with methotrexate (-31.3 *vs*. -20.0).^[58]^ The SELECT-MONOTHERAPY trial showed that at 14 weeks, the ACR20 response rates in RA patients treated with upadacitinib 15 mg and 30 mg as monotherapy were significantly higher than that of methotrexate (68.0%, 71.0% *vs*. 41.0%).^[59]^ The RAJ3 trial showed that at 12 weeks, the ACR20 response rates in RA patients treated with peficitinib 100 mg and 150 mg as monotherapy were significantly higher than that of placebo (57.7%, 74.5% *vs*. 30.7%).^[60]^

The 2024 China RA Guidelines for the management of RA recommended that for patients in persistent remission for at least 6 months may consider reducing the dose of biological disease-modifying antirheumatic drug (bDMARD) /targeted synthetic disease-modifying antirheumatic drug (tsDMARD, including JAK inhibitor) or csDMARD.^[44, 45, 46, 47]^ The 2022 EULAR recommendations recommended that if sustained remission was maintained with bDMARD or tsDMARD (including JAK inhibitors) treatment, the dose may be tapered, but the medication should not be discontinued.^[55]^ The 2021 ACR RA treatment guidelines emphasized that patients with remission for less than 6 months should not discontinue or reduce DMARDs.^[61]^ In addition, the guidelines/consensus recommend that csDMARD should be the first choice for dose reduction or discontinuation. The 2022 EULAR RA recommendation suggested that if JAK inhibitor was used in combination with glucocorticoids, glucocorticoid should be discontinued first before considering dose reduction of JAK inhibitor.^[55]^ The 2021 ACR RA treatment guidelines recommended that for patients receiving MTX in combination with b/tsDMARD, if one medication needed to be discontinued, MTX should be the first choice to reduce or discontinue, rather than b/tsDMARD; at the same time, it was not recommended to discontinue all DMARDs at the same time. If sustained remission is maintained after reducing one drug, gradual discontinuation of that drug may be considered to avoid disease relapse or irreversible damage.^[61]^ Currently, evidence for tapering of JAK inhibitors in RA after achieving treatment targets is still limited. An observational study showed that among patients who achieved remission or low disease activity after one year of continuous tofacitinib treatment, the relapse rate was significantly higher in the discontinuation group compared to the tapering group (0.73 *vs*. 0.44 per patient-year). Moreover, the median relapse-free survival (RFS) was significantly shorter in the discontinuation group (7.0 [95% CI: 2.8–11.2] *vs*. 21.0 months [95% CI: 4.1–37.9]), suggesting that tapering is superior to discontinuation after reaching treatment targets.^[62]^

### Consensus 5

For adult patients with active axSpA who have inadequate response or intolerance to NSAIDs or TNF inhibitors, JAK inhibitors are recommended. For patients whose disease has been stable for ≥ 6 months, dose reduction may be considered (Evidence Level: A).

A Phase III study showed that at week 16, the Assessment of Spondyloarthritis International Society 20% (ASAS20) response rate in adult patients with AS treated with tofacitinib was significantly higher than that of placebo (56.4% *vs*. 29.4%).^[63]^ Another Phase III study showed that at Week 14, the ASAS40 response rate in patients treated with upadacitinib was significantly higher than that of placebo (45% *vs*. 18%).^[64]^ In addition, a Phase II/III trial showed that at Week 16, the ASAS20 response rate in patients with AS treated with ivarmacitinib reached 62.7%.^[65]^ Regarding the use of JAK inhibitors for non-radiographic axial spondyloarthritis (nr-axSpA), upadacitinib is currently the only JAK inhibitor approved by the NMPA for treating adult patients with active nr-axSpA. The SELECT-AXIS 2 trial confirmed that the ASAS40 response rate in nr-axSpA patients treated with upadacitinib was significantly higher than with placebo (45% *vs*. 23%).^[66]^

Currently, there is no clinical evidence on the impact of JAK inhibitor dose reduction or discontinuation on disease control in patients with axSpA. Previous consensus has indicated that patients with SpA need to maintain low disease activity or remission for at least 6 months before considering a dose reduction of targeted therapies.^[67,68]^ Given the current lack of specific guidelines for tapering or discontinuing JAK inhibitors, this consensus recommends that axSpA patients receiving JAK inhibitor treatment may consider a dose reduction if their disease has been stable for ≥ 6 months, in order to ensure sustained effective disease control.

### Consensus 6

JAK inhibitors are recommended for adult patients with PsA who have inadequate response or intolerance of one or more DMARDs; dose reduction may be considered in patients who have maintained sustained remission for ≥1 year (Evidence Level: A).

A Phase III trial of adult polyarticular PsA in China showed that at 3 months of treatment, the ACR50 response rate of the tofacitinib group was significantly higher than that of the placebo group (38.2% *vs*. 5.9%).^[69]^ In addition, the SELECT-PsA 1 trial showed that at 12 weeks of treatment, the ACR20 response rates of the upadacitinib 15 mg group and 30 mg group were significantly higher than of the placebo group (15 mg: 70.6%, 30 mg: 78.6% *vs*. 36.2%).^[70]^

Previous guidelines have indicated that PsA patients who achieved sustained remission may be considered for a reduction in the DMARD dosage.^[71]^ Based on these principles, this consensus suggests that PsA patients treated with JAK inhibitors may be considered for a dose reduction after maintaining sustained remission for at least one year.

### Consensus 7

For patients with active JIA aged 2 years and older who have inadequate response or intolerance to csDMARDs/bD-MARDs, JAK inhibitors are recommended. For patients who have achieved sustained remission for ≥ 1 year, dose reduction may be considered. It is recommended that children with polyarticular or oligoarticular JIA should continue the treatment for 2 years after achieving clinical remission (Evidence Level: A).

A Phase III study demonstrated that in the treatment of JIA, the time to disease recurrence in the baricitinib group was significantly longer than that in the placebo group (hazard ratio [HR] 0.241, 95% CI: 0.128–0.453).^[72]^ In addition, multiple studies in JIA patients have shown that the risk of recurrence after discontinuation of DMARDs was high, with a recurrence rate of 31.7% at 1 year, and the recurrence rate increased to 54.7% at 2 years.^[73,74]^ A retrospective study showed that in patients with polyarticular and oligoarticular JIA, those who continued the use of biological agents for more than 2 years after disease remission had a longer duration of remission after discontinuation (18.64 ± 3.3 *vs*. 11.51 ± 2.7 months).^[75]^ A discontinuation study of tofacitinib also confirmed that after 18 weeks of continuous treatment and achieving remission, the recurrence rate at week 44 was significantly higher in the discontinuation group than in the continued treatment group (53% *vs*. 29%).^[76]^ This evidence suggests that extending treatment duration after remission in JIA helps maintain the remission after drug discontinuation. Previous guidelines recommended that JIA patients who have achieved clinical remission for at least 1 year may gradually reduce the dose or frequency of csDMARDs and biologics (including JAK inhibitors); for children with polyarticular and oligoarticular JIA, it is recommended to continue treatment for 2 years after clinical remission was achieved.

### Consensus 8

JAK inhibitors are recommended for the treatment of new-onset or recurrent GCA in adults (Evidence Level: B).

A prospective study showed that among recurrent GCA patients treated with baricitinib, 93% (13/14) achieved remission of glucocorticoids by week 52.^[77]^ Results from another Phase III trial showed that the sustained remission rate at week 52 in patients with new-onset or recurrent GCA treated with upadacitinib was significantly higher than that of the placebo group (46.4% *vs*. 29.0%).^[78]^ Furthermore, a retrospective study showed that at 3 and 6 months of treatment, GCA patients treated with JAK inhibitors (baricitinib or tofacitinib) had significant reductions in both CRP levels and daily prednisolone dose.^[79]^ Additionally, a real-world study showed that after a median follow-up of 11 months, the clinical remission rate in recurrent GCA patients treated with JAK inhibitors (including baricitinib, tofacitinib, and upadacitinib) was 57%, with a complete remission rate of 46%.^[80]^

### Consensus 9

JAK inhibitors are recommended for the treatment of moderate-to-severe AD. Patients prioritized for JAK inhibitors include: (1) those requiring rapid relief of disease symptoms; (2) those unwilling to undergo subcutaneous injections or phototherapy; (3) those who have developed immune drift from the use of biologic agents. After achieving an adequate response following 3 to 6 months of standardized treatment, the dose could be reduced for maintenance. If discontinuation is considered, an intermittent dosing regimen, such as once every other day or twice weekly, is recommended as gradual tapering (Evidence Level: A).

Multiple studies have confirmed the significant therapeutic efficacy of JAK inhibitors in moderate to severe AD. The Level Up study showed that at Week 16 of treatment, upadacitinib treatment in adult/adolescent patients with moderate to severe AD achieved significant improvement in both Eczema Area and Severity Index (Eczema Area and Severity Index, EASI) with ≥ 90% improvement (EASI 90) and the Worst Pruritus Numerical Rating Scale (Worst Pruritus Numerical Rating Scale, WP-NRS) returning to 0 or 1 part (WP-NRS 0/1) with significantly higher response rates compared to dupilumab (19.9% *vs*. 8.9%).^[81]^ The JADE MONO-1 study showed that at week 12, response rates for Investigator Global Assessment (IGA) (24% and 44% *vs*. 8%) and EASI 75 (40% and 63% *vs*. 12%) in the abrocitinib 100 mg and 200 mg groups, respectively, were significantly higher than that of the placebo group.^[82]^ An international, multicenter Phase III study of ivarmacitinib also showed that at week 16, rates of achieving IGA 0/1 with a ≥ 2-point improvement from baseline (36.3% and 42.0% *vs*. 9.0%) and EASI 75 (54.0% and 66.1% *vs*. 21.6%) were significantly higher in the 4 mg and 8 mg groups, respectively, compared to placebo.^[83]^

JAK inhibitors have been demonstrate to have rapid onset of action in the treatment of AD, particularly in relieving pruritus. A meta-analysis showed that JAK1 inhibitors (abrocitinib, upadacitinib) began to demonstrate efficacy as early as week 2 of treatment, with significantly higher EASI 75 (RR 3.76, 95% CI: 3.21–4.41) and EASI 90 (RR 2.59, 95% CI: 1.82–3.69) response rates compared to the control group (placebo or IL-4 inhibitors).^[84]^ The Heads-Up study showed that at week 1, the mean improvement in WP-NRS in patients with moderate-to-severe AD treated with upadacitinib was significantly superior to IL-4 inhibitors (31.4% *vs*. 8.8%).^[85]^ The LEVEL-UP trial showed that at week 2, the response rates of WP-NRS 0/1 (7.7% *vs*. 1.3%) and EASI 75 (26.7% *vs*. 8.2%) in adults and adolescents with moderate-to-severe AD treated with upadacitinib were significantly better than those treated with IL-4 inhibitors.^[81]^ The JADE-DARE trial showed that at week 2, the proportion of patients achieving a ≥ 4-point improvement in the Peak Pruritus Numerical Rating Scale (PP-NRS) in moderate-to-severe adult AD treated with abrocitinib was significantly higher than that in the IL-4 inhibitor group (48% *vs*. 26%).^[86]^ This consensus recommended JAK inhibitors for patients with AD who require rapid relief of symptoms.

According to statistics, in addition to rapid relief of pruritus and safety, patients prefer daily oral administration (preference weight 0.796, 95% CI: 0.609–0.984).^[87]^ Previous consensuses/guidelines indicated that AD patients who were unwilling to receive subcutaneous injections and were able to take oral medication on time prefered JAK inhibitors;^[48]^ phototherapy is generally performed in clinics, and its feasibility is constrained by travel distance, study or work commitments, time arrangements, and medical insurance.^[88]^ Therefore, this consensus recommends that AD patients who are unwilling to receive subcutaneous injections or phototherapy may choose JAK inhibitors.

Studies showed that IL-4/13 inhibitors used for AD carried the risk of secondary PsO or inflammatory arthritis.^[89,90]^ JAK inhibitors can block the signal transduction pathways of multiple cytokines, including IL-4, IL-13, IL-22, IFN-γ, and thymic stromal lymphopoietin (TSLP), and show certain advantages in AD patients who have experienced immune drift after treated with biological agents. This consensus recommends that AD patients who have experienced immune drift with biological agents should choose JAK inhibitors.

A Phase III dose-tapering study showed that in patients with moderate-to-severe AD, among those who initiated a dose-reduction maintenance regimen at Week 12 of abrocitinib treatment, the salvage therapy rate at Week 40 was significantly lower than that in the discontinuation (placebo) group (39.2% *vs*. 76.4%).^[91]^ In addition, JAK inhibitors can achieve dose-reduction goals by extended dosing intervals: a prospective observational cohort study showed that in elderly patients with moderate-to-severe AD receiving upadacitinib using a dose-reduction pattern (26.0% of patients on once every 3 days, 72.0% on once every 2 days), the EASI 75 and EASI 50 response rates reached 46.0% and 92.0%, respectively, at month 12, and no patients discontinued treatment due to insufficient efficacy.^[92]^ Previous consensuses had pointed out that in children and adolescents with AD receiving systemic therapy, a gradual reduction in treatment intensity could be considered after months of complete or near-complete clearance of skin lesions.^[93]^ After 3 to 6 months of standardized JAK inhibitor treatment and achieving a sufficient response, an intermittent dosing regimen such as once every other day or twice a week could be adopted as an attempt to taper or discontinue the medication.^[94]^

### Consensus 10

JAK inhibitors are recommended for the treatment of patients with moderate to severe AA. To reduce the risk of recurrence, treatment should be maintained for at least 6 to 12 months after complete hair regrowth before considering gradual dose reduction (Evidence level: A).

The BRAVE-AA1/AA2 trial results showed that at week 36 of treatment, the SALT score ≤ 20 response rates of the baricitinib 4 mg group and 2 mg group in the treatment of adults with severe AA were significantly higher than those of the placebo group (AA1: 38.8%, 22.8% *vs*. 6.2%; AA2: 35.9%, 19.4% *vs*. 3.3%).^[95]^ A Phase IIb/III trial showed that at week 24, the SALT score ≤ 20 response rates of the ritlecitinib 50 mg group and 30 mg group in the treatment of adult and adolescent patients with severe AA were significantly higher than that of the placebo (23%, 14% *vs*. 2%).^[96]^ A Phase II study showed that at week 24, the change in SALT score from baseline in patients with moderate to severe AA treated with ivarmacitinib 4 mg and 8 mg was significantly greater than that of the placebo group (-56.11%, -51.01% *vs*.-19.87%).^[97]^ Another Phase III study showed that at week 24, the SALT score ≤ 20 response rates in patients with severe AA treated with ivarmacitinib 4 mg and 8 mg were significantly higher than that of the placebo (34.9%, 40.6% *vs*. 9.0%).^[98]^

A meta-analysis showed that the recurrence rate in patients with AA treated with JAK inhibitors (including tofacitinib, ruxolitinib, baricitinib, ritlecitinib, and brepocitinib) was 54% (95% CI: 39%–69%), and recurrence was mainly associated with the discontinuation of JAK inhibitors.^[99]^ Based on this, to maintain the effect of hair regrowth, it is recommended that patients with AA who tolerate JAK inhibitors and achieve a good treatment response should continue treatment; and after complete hair regrowth, maintenance treatment should continue for at least 6 to 12 months before considering a switch to a maintenance treatment regimen or discontinuation.^[100]^

### Consensus 11

TYK2 inhibitors are recommended for adult patients with moderate-to-severe plaque PsO who are candidates for systemic therapy or phototherapy (Evidence Level: A).

The results of the POETYK PSO-1 trial showed that at Week 16, deucravacitinib (a TYK2 inhibitor), when used in patients with moderate-to-severe plaque PsO, achieved a significantly higher Psoriasis Area and Severity Index (PASI) 75 (improvement of ≥ 75%) response rate compared to placebo and apremilast (58.4% *vs*. 12.7% and 35.1%, respectively).^[101]^

### Consensus 12

JAK inhibitors are recommended for adult patients with moderate-to-severe active UC who have an inadequate response or are intolerant of TNF inhibitors (Evidence Level: A).

A Phase III trial showed that patients with UC receiving tofacitinib induction therapy (10 mg twice daily [BID]) had a significantly higher clinical remission rate at Week 8 compared to the placebo group (18.5% *vs*. 8.2%); during the maintenance phase, patients receiving 5 mg or 10 mg BID achieved significantly higher clinical remission rates at Week 52 compared to the placebo group (34.3% and 40.6% *vs*. 11.1%).^[102]^ A multi-center Phase III trial showed that patients with moderate-to-severe UC receiving upadacitinib induction therapy (45 mg once daily [QD]) had a significantly higher clinical remission rate at Week 8 compared to the placebo group (33% *vs*. 4%). During the maintenance phase, clinical remission rates at Week 52 for patients receiving 15 mg or 30 mg QD were also significantly superior to the placebo group (40.4% and 53.6% *vs*. 10.8%).^[103,104]^ A Phase II RCT study showed that at Week 8, the clinical remission rates of the ivarmacitinib 8 mg QD, 4 mg BID, and 4 mg QD groups were all significantly higher than that of the placebo group (22.0%, 24.4%, and 24.4% *vs*. 4.9%); after the initial 8-week treatment period, ivarmacitinib was continued for a further 8-week extension treatment, and mean Mayo scores continued to decrease or remained stable.^[105]^

For patients with UC with an insufficient response to induction therapy, extending the induction therapy cycle may be considered.^[46,106]^ The OCTAVE Open extension study showed that patients with UC who did not respond to initial induction therapy achieved a 52.2% clinical response rate at week 16 after receiving tofacitinib 10 mg BID for extended induction therapy.^[107]^ Similarly, an upadacitinib extended induction trial showed that patients with moderate-to-severe UC who did not respond to 8 weeks of induction therapy achieved a 59.1% clinical remission rate at Week 16 after continuing upadacitinib 45 mg for extended induction.^[108]^

### Consensus 13

JAK inhibitors are recommended for adult patients with moderate-to-severe active CD who have an inadequate response or are intolerant to TNF inhibitors (Evidence Level: A).

Two Phase IIb RCT trials showed that in patients with moderate-to-severe CD receiving tofacitinib induction therapy (5 mg and 10 mg twice daily), the clinical remission rates at Week 8 were numerically higher than those of the placebo group (43.5% and 43.0% *vs*. 36.7%); after entering the 26-week maintenance phase, the “Clinical Response 100” (defined as Crohn’s Disease Activity Index [CDAI] reduction from baseline of ≥ 100 points) and clinical remission rates remained numerically higher than those of the placebo group (39.5% and 55.8% *vs*. 38.1%).^[109]^ The U-EXCEL trial demonstrated that for patients with moderate-to-severe CD receiving upadacitinib 45 mg once daily induction therapy, the CDAI clinical remission rate (49.5% *vs*. 29.1%) and endoscopic response rate (45.5% *vs*. 13.1%) at week 12 were both significantly higher than those of the placebo group; during the maintenance phase using 15 mg or 30 mg once daily doses, the CDAI clinical remission rates (37.3% and 47.6% *vs*. 15.1%) and endoscopic response rates (27.6% and 40.1% *vs*. 7.3%) at week 52 were also significantly superior to the placebo group.^[110]^ The LATTICE-CD trial showed that in patients with moderate-to-severe CD receiving deucravacitinib 3 mg induction therapy, the endoscopic response rate at week 12 was higher than that of the placebo group (23.3% *vs*. 8.3%), and the clinical remission rate was numerically superior (31.4% *vs*. 28.3%).^[111]^ Furthermore, the U-EXCEL trial showed that for patients with moderate-to-severe CD with no response to initial induction, after receiving upadacitinib 45 mg extended induction therapy, the clinical remission rate and endoscopic response rate at Week 24 reached 40.5% and 37.8%, respectively, suggesting that for patients with insufficient response to induction therapy, the duration of JAK inhibitor induction therapy may be extended.^[110]^

### Consensus 14

JAK inhibitors are conditionally recommended for the treatment of IMIDs such as hand eczema, vitiligo, SLE, dermatomyositis, and Behçet’s disease (Evidence Level: B).

#### (I) Hand eczema

A prospective study showed that the mean time to achieve a Hand Eczema Severity Index (HECSI) improvement of ≥ 90% (HECSI-90) in patients with refractory chronic hand eczema treated with tofacitinib was 4 weeks.^[17]^ A prospective observational cohort study showed that at Week 16, the HECSI-75 response rate in patients with atopic dermatitis combined with contact hand eczema treated with upadacitinib was 59.3%, and the skin lesion clearance rate was 74.1%.^[112]^ Another real-world study showed that in patients with refractory hand eczema treated with abrocitinib, the HECSI-75 response rate at Week 4 was 50% (6/12), and the HECSI-90 response rate at Week 16 was 100%.^[113]^

#### (II) Vitiligo

A RCT trial showed that at Week 24, the overall clinical efficacy rate of tofacitinib combined with phototherapy in the treatment of vitiligo patients was significantly higher than that of phototherapy alone (84.1% *vs*. 65.6%).^[18]^ An RCT study showed that at Week 36, the mean change from baseline in the Vitiligo Area Scoring Index (VASI) for baricitinib combined with phototherapy in the treatment of severe active non-segmental vitiligo was significantly superior to placebo (-44.8% *vs*. -9.2%).^[114]^ A multicenter Phase II RCT trial showed that at Week 24, the mean changes from baseline in the Facial-Vitiligo Area Scoring Index (F-VASI) for upadacitinib 11 mg and 22 mg in patients with non-segmental vitiligo were significantly higher than those of placebo (-35.63% and -33.96% *vs*. 14.36%).^[115]^ Another Phase IIb RCT study showed that at Week 24, the mean changes from baseline in F-VASI for ritlecitinib 50 mg and 30 mg monotherapy in adult non-segmental vitiligo patients were significantly higher than those of placebo (-18.5% and-14.6% *vs*. 2.1%).^[116]^

#### (III) Sjögren’s syndrome

In the treatment of Sjögren’s syndrome, evidence for the use of JAK inhibitors still lacks support from large-sample clinical studies. However, the results of a preliminary exploratory study showed that at Month 6, the response rate of baricitinib in patients with moderate-to-severe Sjögren’s syndrome (defined as reaching the criterion of “at least a 3-point improvement” in the EULAR Sjögren’s Syndrome Patient Reported Index [ESSPRI]) was as high as 88.9%.^[19]^

#### (IV) SLE

The SLE-BRAVE-I trial showed that at Week 52, the response rate of the Systemic Lupus Erythematosus Responder Index-4 (SRI-4) in patients with active SLE treated with baricitinib was significantly superior to placebo (57% *vs*. 46%).^[20]^ Another Phase II RCT study showed that at Week 24, the response rate of the upadacitinib group achieving “SRI-4 and glucocorticoid dose ≤ 10 mg/d” was significantly higher than that of placebo (54.8% *vs*. 37.3%).^[117]^ Currently, studies on tofacitinib in SLE have only completed Phase I safety assessments and have not yet included efficacy endpoints.^[118]^ The above evidence suggested that JAK inhibitors might provide a new treatment direction for the gradual tapering of glucocorticoids in SLE patients, but the safety of long-term use still needs further validation through clinical studies.

#### (V) Vasculitis

A prospective observational study showed that adult patients with Takayasu arteritis receiving tofacitinib combined with glucocorticoids had significantly higher complete remission rates at Months 6 and 12 compared to the methotrexate combined with glucocorticoid group (85.19% *vs*. 61.54%; 88.46% *vs*. 56.52%).^[21]^ A preliminary exploratory study showed that tofacitinib treatment was effective in patients with antineutrophil cytoplasmic antibody (ANCA)-associated vasculitis, with a complete remission rate of 90% (9/10) and a partial remission rate of 10%.^[119]^

#### (VI) Dermatomyositis

A prospective study showed that treatment with tofacitinib in patients with newly diagnosed anti-melanoma differentiation-associated gene 5 (MDA5) antibody-positive dermatomyositis achieved a clinical response rate of 71.4% (10/14), with a median Total Improvement Score (TIS) of 43.75.^[22]^ A prospective study showed that at Week 12, treatment with tofacitinib extended-release tablets in patients with refractory active dermatomyositis achieved a 100% (10/10) response rate in disease activity improvement.^[120]^ A prospective study showed that at Week 4, treatment with baricitinib in patients with refractory juvenile dermatomyositis achieved a 50% (2/4) response rate in ACR/EULAR fatigue improvement, and at Week 8, significant improvement in Manual Muscle Testing (MMT).^[121]^ Another prospective study showed that at Week 12, treatment with baricitinib in adult dermatomyositis patients achieved a 75.0% (9/12) response rate for a 40% reduction in the Cutaneous Dermatomyositis Disease Area and Severity Index (CDASI) score.^[122]^ These studies indicated that JAK inhibitors could alleviate fatigue symptoms and disease activity in dermatomyositis patients, bringing substantial skin improvement.

#### (VII) SSc

A preliminary RCT study showed that at Weeks 26 and 52, the modified Rodnan skin score (mRSS) in tofacitinib-treated SSc patients was significantly lower than that in the methotrexate group (12.00 *vs*. 21.00; 10.00 *vs*. 20.00).^[23]^ A Phase I/II RCT study showed that at Week 24, the Physician Global Assessment (PGA) score in tofacitinib-treated patients with early diffuse cutaneous SSc improved significantly more from baseline than that in the placebo group (-1.5 *vs*. 0.5).^[123]^

#### (VIII) Hidradenitis suppurativa

A Phase II RCT study reported that at week 12, the clinical response rate (HiSCR50) of upadacitinib in hidradenitis suppurativa was significantly superior to placebo (38.3% *vs*. 25.0%).^[24]^ Another case report showed that tofacitinib treatment in patients with hidradenitis suppurativa who were previously non-responsive to biologics resulted in ulcer healing and pain reduction.^[124]^

#### (IX) Behçet’s disease (including intestinal Behçet’s disease)

A few studies have demonstrated the positive effects of JAK inhibitors in the treatment of Behçet’s disease. A preliminary exploratory study demonstrated that after treatment with upadacitinib for refractory Behçet’s disease, the overall Behçet’s Disease Current Activity Form (BDCAF) score improved significantly from baseline following a median follow-up of 8 months (median score: 5 *vs*. 0).^[25]^ Another case report showed that in a patient with intestinal Behçet’s disease unresponsive to systemic glucocorticoids, sulfasalazine, and TNF inhibitors, symptoms of abdominal pain improved rapidly after upadacitinib treatment, and CRP, erythrocyte sedimentation rate (ESR), and fecal calprotectin returned to normal, with complete ulcer healing observed at the 12-week follow-up.^[125]^

### Consensus 15

JAK inhibitor ointments/creams are conditionally recommended as a treatment option for patients with AA, AD, PsO, vitiligo, and hand eczema who have inadequate response to conventional therapy (Evidence Level: B).

The current NMPA marketing progress and clinical application of JAK inhibitor ointments are detailed in Table 5.

**Table 5:** Overview of topical JAK inhibitors

Medication	Regulatory status	Indication	Study type	Clinical results
Ruxolitinib cream	Under regulatory review	AD	Phase III RCT^[218,219]^	At week 8, IGA 0/1 response rates (with ≥ 2-point improvement from baseline) significantly higher with ruxolitinib 0.75% and 1.5% cream versus vehicle (Study 1: 50.0%, 53.8% *vs*. 15.1%; Study 2: 39.0%, 51.3% *vs*. 7.6%).
Ruxolitinib cream	N/A	AA	Phase II study^[220]^	At week 24, the SALT50 response rates for ruxolitinib 1.5% cream was 50%.
Ruxolitinib cream	N/A	PsO	Pilot study^[221]^	Ruxolitinib 1% and 1.5% creams improved lesion thickness, erythema, and lesion area; overall lesion score improvement was greater with 1% cream than with vehicle (54% *vs*. 32%).
Ruxolitinib cream	Under regulatory review	Vitiligo	Phase III RCT^[222]^	At week 24, Ruxolitinib 1.5% cream achieved significantly higher F-VASI 75 response compared to vehicle (TRUE-V1 trial: 29.8% *vs*. 7.4%; TRUE-V2 trial: 30.9% *vs*. 11.4%).
Tofacitinib oint- ment	Under regulatory review	AA	Small-sample pilot study^[223]^	At week 24, tofacitinib 2% ointment produced a mean SALT score reduction of 34.6%.
Tofacitinib ointment	N/A	AA	Prospective intrasubject controlled study^[224]^	At week 24, the SALT50 response rate with tofacitinib 2% ointment reached 40%, and the hair-pull test positivity rate was significantly lower than that with vehicle (10% *vs*. 86.7%).
Ivarmacitinib ointment	Under Regulatory Review	AD	Phase III RCT^[225]^	At week 8, ivarmacitinib 0.5% and 1% ointment achieved significantly higher IGA 0/1 response rates (≥ 2-point improvement from baseline) than vehicle (21.3%, 26.2% *vs*. 10.6%), as well as significantly higher EASI-75 response rates (42.6%, 45.1% *vs*. 17.9%).
Delgocitinib cream	N/A	AD	Phase III study^[226]^	At week 4, delgocitinib 0.5% cream resulted in a significantly greater improvement in EASI score from baseline compared with vehicle (−44.3% *vs*. 1.7%).
Delgocitinib cream	N/A	Hand eczema	Phase IIb study^[227]^	At week 16, the IGA 0/1 response rates (≥ 2-point improvement from baseline) with delgocitinib 8 mg/g and 20 mg/g cream were significantly higher than those with vehicle (36.5%, 37.7% *vs*. 8.0%).

AD, atopic dermatitis; AA, alopecia areata; EASI, Eczema Area and Severity Index; F-VASI, Facial Vitiligo Area Scoring Index; IGA, Investigator’s Global Assessment; N/A, not applicable; PsO, psoriasis; RCT, randomized controlled trial; SALT, Severity of Alopecia Tool.

### Consensus 16

When JAK inhibitors are used in combination with biologics or immunosuppressants, the risk of serious adverse events should be thoroughly assessed. In patients receiving concomitant glucocorticoid therapy, glucocorticoids may be considered for gradual tapering or discontinuation after achieving stable disease (Evidence Level: B).

A retrospective study showed that the treatment of refractory CD patients with upadacitinib in combination with ustekinumab achieved clinical remission in 83.3% (5/6) of patients with active refractory CD during a median follow-up period of 10 months. Among the 2 patients with extraintestinal manifestations of joint pain, joint symptoms improved. No serious adverse events, such as herpes zoster, thromboembolism, major adverse cardiovascular events (MACE), or malignancies, were observed during the follow-up period.^[126]^ Another case report included 6 refractory PsO and PsA patients treated with a combination of JAK inhibitors and IL-17 or IL-23 inhibitors. The results showed that all patients achieved skin improvement or reached minimal disease activity (MDA). During the follow-up period, only 1 patient developed bronchitis, and no other adverse events were reported.^[127]^ In addition, JAK inhibitors in combination with vedolizumab have shown certain therapeutic efficacy in the treatment of refractory UC patients in some exploratory studies.^[128, 129, 130]^ However, the long-term safety of this regimen requires validation with high-level evidence. The SELECTION trial, which analyzed the treatment of patients with moderate-to-severe UC with filgotinib in combination with immunosuppressants such as azathioprine, showed that the clinical remission rates at Weeks 10 and 58 were similar between the combination treatment group and the monotherapy group, and no significant difference in the incidence of adverse events was observed.^[131]^ It should be particularly noted that the combination of JAK inhibitors with biological agents (*e.g*., IL-17/IL-23 inhibitors, vedolizumab) or immunosuppressants may increase the risk of herpes zoster, tuberculosis (TB) reactivation, hematologic abnormalities (*e.g*., neutropenia), and malignancies. Therefore, if a combination therapy regimen is used, the risk of adverse events should be fully assessed.

A retrospective study showed that at Month 6 of treatment, the mean change in oral glucocorticoid dosage in the JAK inhibitor group was significantly higher than that in the bDMARDs group (-1.26 mg/d vs-0.71 mg/d), and the bD-MARDs group had a higher risk of requiring glucocorticoid rescue therapy (RR 2.66, 95% CI: 1.88–2.74).^[132]^ A meta-analysis indicated that in patients with moderate-to-severe UC treated with tofacitinib, the clinical remission rates with discontinuation of oral glucocorticoids at week 8, weeks 12–16, month 6, and month 12 were 38.4%, 44.3%, 33.6%, and 31%, respectively.^[133]^ JAK inhibitors can assist in reducing the dosage of oral glucocorticoids and shortening the duration of use. Therefore, for patients with IMIDs who have persistently active disease and find it difficult to discontinue glucocorticoids, combination with JAK inhibitors can facilitate the gradual reduction and discontinuation of oral glucocorticoids.

### Consensus 17

Prior to the use of JAK inhibitors, the potential risks and benefits should be adequately assessed, including thromboembolic events, severe cardiovascular events, and malignant neoplasms, and safety monitoring should be conducted. The monitoring parameters and frequency should be adjusted according to clinical needs. However, the potential differences in the types and incidence rates of adverse events among different JAK inhibitors should be noted (Evidence Level: B) (Part 1).

#### (I) Infection

JAK inhibitors may reduce the body’s resistance to pathogens such as bacteria and viruses, thereby increasing the risk of various infections, including upper respiratory tract infections, lung infections, herpes zoster, mycobacterium tuberculosis, and hepatitis virus infections.^[134,135]^ A meta-analysis including 32 RCT studies (involving 11, 917 patients immune-mediated inflammatory skin diseases) showed that the incidence of severe infections in the JAK inhibitor group and the control group (including TNF inhibitors, IL-4 inhibitors, phosphodiesterase 4 inhibitors, inhibitors, and placebo) was 0.62% and 0.51%, respectively, and the use of JAK inhibitors was not significantly associated with the risk of severe infections in patients with immune-mediated inflammatory skin diseases (RR 0.68, 95% CI: 0.43–1.07).^[136]^ Another multicenter real-world study showed that the risk of infection (HR 0.97, 95% CI: 0.75–1.24) and the risk of infection-related hospitalization (HR 0.59, 95% CI: 0.27–1.05) were similar between JAK inhibitors and TNF inhibitors in patients with inflammatory bowel disease (IBD).^[137]^

The above results suggested that JAK inhibitors might increase the risk of infection or reactivation of existing infection. When patients with IMIDs developed symptoms such as fever, cough, or dyspnea, appropriate symptomatic treatment should be initiated, and patients should be closely monitored. It is recommended to discontinue the JAK inhibitor until clinical symptoms have completely resolved.

#### (II) Cardiovascular events and thrombosis

Treatment with JAK inhibitors is associated with mild increases in total cholesterol, high density lipoprotein cholesterol (HDL-C), and low density lipoprotein cholesterol (LDL-C).^[138]^ Treatment with JAK inhibitors increases the risk of venous thromboembolism (VTE), including pulmonary embolism (PE) and deep venous thrombosis (DVT).^[139]^ However, a meta-analysis published in JAMA Dermatology showed that JAK inhibitor treatment for immune-mediated inflammatory skin diseases does not increase the risk of MACE or all-cause mortality.^[140]^ A study by Yates *et al* showed that the odds ratios for the incidence of VTE, PE, and DVT in patients receiving JAK inhibitor treatment were 0.68 (95% CI: 0.36–1.29), 0.44 (95% CI: 0.28–0.70), and 0.59 (95% CI: 0.31–1.15), respectively, and did not confirm the current concerns regarding JAK inhibitors increasing the risk of VTE.^[141]^ Other observational studies also found that the incidence of VTE in patients receiving JAK inhibitors and bDMARDs treatment was comparable, with incidence rates of 0.29 (95% CI: 0.13–0.54) /100 patient-years and 0.33 (95% CI: 0.24–0.45) /100 patient-years, respectively.^[142]^ In addition, a meta-analysis of 26 RCT studies (involving 11, 799 RA patients) showed that, compared with placebo, tofacitinib (OR 0.63, 95% CI: 0.26–1.54), baricitinib (OR 1.21, 95% CI: 0.51–2.83), upadacitinib (OR 3.29, 95% CI: 0.59–18.44), and peficitinib (OR 0.43, 95% CI: 0.07–2.54) did not show a significant difference in the overall risk of cardiovascular events.^[143]^

Before initiating or continuing JAK inhibitors, the risk of cardiovascular events and the treatment benefit should be fully assessed;^[144, 145, 146]^ JAK inhibitors should be used with caution in patients with a current or past smoking history, in combination with other cardiovascular risk factors, or with a risk of VTE. At the same time, patients should be fully informed of the typical symptoms of severe cardiovascular events and the correct response measures; if cardiovascular events appear clinically during treatment, the JAK inhibitor should be discontinued immediately.

#### (III) Malignancy

The overall incidence of malignant tumors in the treatment of IMIDs with JAK inhibitors is 0.89/100–1.26/100 patient-years.^[147,148]^ A meta-analysis showed that the incidence of malignant tumors with JAK inhibitors (tofacitinib) (OR 2.39, 95% CI: 0.50–11.5) was similar to that of other bDMARDs (including TNF inhibitors, abatacept, rituximab, and tocilizumab), with no increased risk of tumors.^[149]^ Another retrospective study showed that JAK inhibitors, compared with IL-4 inhibitors, did not increase the risk of malignant tumors (HR 1.29, 95% CI: 0.79–2.10).^[150]^

Before initiating JAK inhibitor treatment, the risks and benefits should be carefully weighed, especially with particular caution for the following patients: patients with known malignant neoplasms (excluding successfully treated non-melanoma skin cancer), patients with newly diagnosed malignant neoplasms during treatment, and patients with a current or past smoking history. During treatment, close monitoring and vigilance for malignant neoplasms should be maintained. If a malignant neoplasm is diagnosed, JAK inhibitor treatment should be discontinued immediately. A comprehensive analysis of the medical condition should be conducted, and if necessary, a multidisciplinary team should be involved to evaluate the subsequent management and treatment strategy. In addition, the safety of JAK inhibitors in patients with a history of malignant neoplasms is not yet well established. Given that such patients are often excluded from clinical trials, the long-term drug safety still depends on long-term real-world registry follow-up data for further confirmation.^[151]^

#### (IV) Other common adverse events

Common adverse events associated with JAK inhibitors mainly include hematologic system abnormalities, liver and kidney function abnormalities, and gastrointestinal reactions. Adverse reactions of the hematologic system are associated with the JAK2 pathway mediated by hematopoietic cytokines. Erythropoietin mainly exerts its effects through the JAK2 signaling pathway, and thus suppression of JAK2 may induce neutropenia, anemia, and thrombocytopenia.^[152,153]^ Lymphopenia (0.1%–2.4%), thrombocytopenia (1.8%–3.2%), neutropenia (0.2%–5%), and anemia (0.1%–2.4%) are common hematological abnormalities,^[154]^ and the risk of occurrence was dose-dependent, indicating the necessity of regular laboratory tests.^[155,156]^ A real-world study showed that pan-JAK inhibitors (tofacitinib and baricitinib) were independent risk factors for medication discontinuation in RA patients (HR 4.49, 95% CI: 1.05–19.23); the discontinuation rates due to adverse events for tofacitinib, baricitinib, and JAK1 inhibitors (upadacitinib, filgotinib) were 21.7%, 34.1%, and 2.9%, respectively, and the incidence of hematologic adverse events for tofacitinib, baricitinib, and JAK1 inhibitors were 6.7%, 8.7%, and 0%, respectively.^[157]^ A Phase III study showed that the incidence of anemia in RA patients treated with ivarmacitinib 4 mg and 8 mg was lower than that of the placebo (6.3%, 7.4% *vs*. 11.7%).^[54]^ Therefore, the monitoring frequency for hematologic system abnormalities should be increased for pan-JAK inhibitors.

In JAK inhibitor-related studies, there were reports of transient increases in aspartate aminotransferase (AST) and alanine aminotransferase (ALT) levels, and serious adverse events associated with increased AST and ALT were not common. In addition, there were reports of elevated creatinine, but these were unrelated to renal injury.^[158,159]^

Abdominal pain (4.0%), diarrhea (3.6%), and nausea and vomiting (3.6%) are common gastrointestinal adverse reactions associated with JAK inhibitors. The underlying mechanism remains unknown and may be related to the suppression of cytokines such as IL-6 and IL-10, thereby reducing the neutralization capacity of IgA against endotoxins and pathogens in the intestines.^[160,161]^ A small number of case reports of gastrointestinal perforation have been reported in patients receiving JAK inhibitors, with an incidence of (1.29–5) /1000 patient-years.^[162]^ In addition, reports of gastrointestinal adverse events associated with tofacitinib had increased significantly (IC025 2.0);^[163]^ regulatory authorities therefore issued warning regarding the risk of diverticulitis and perforation with baricitinib in August 2020.^[164]^ It is recommended to promptly assess patients with new-onset abdominal pain to avoid gastrointestinal perforation as early as possible. Laboratory monitoring before and after JAK inhibitor treatment is detailed in Table 6.

**Table 6:** Laboratory monitoring before and during treatment with JAK inhibitors^[94,228, 229, 230, 231]^

Laboratory parameters	Baseline	≤ 3 Months (Initial period)	> 3 Months or as clinically indicated	Criteria for treatment interruption or discontinuation
ALC	√	√	√	< 0.5×109/L
ANC	√	√	√	< 1×109/L
Hb	√	√	√	< 80 g/L
Liver and renal function	√	√	√	Suspected drug-induced liver injury
Coagulation function	√	√	√	—
Lipid profile	—	√	√	—
Tuberculosis screening	√	—	√	Active tuberculosis
Viral hepatitis screening	√	—	√	Active hepatitis
Herpes zoster	√	—	√	Herpes zoster reactivation

Laboratory monitoring recommendations are primarily based on individual product labels. “—” indicates that routine testing is not required. ALC, absolute lymphocyte count; ANC, Absolute neutrophil count; Hb, Hemoglobin.

### Consensus 18

Medication in special populations: (1) JAK inhibitors should be avoided in pregnant and lactating women, and medication should be discontinued at least 1 month prior to pregnancy planning (or conception); (2) elderly patients receiving JAK inhibitors should be monitored for infection, cardiovascular events, and thromboembolic risk; (3) JAK inhibitors should be used with caution in patients with severe hepatic or renal impairment; (4) live attenuated vaccines are not recommended for patients with rheumatic disorders; in patients receiving inactivated vaccines, JAK inhibitors do not need to be discontinued; (5) for patients undergoing major surgery, JAK inhibitors should be discontinued 3 days prior to surgery, and medication may be resumed after surgery if postoperative wound healing is satisfactory and there is no surgical site or systemic infection (Evidence level: B).

#### (I) Pregnant and lactating women

Research reports have described pregnancy outcomes associated with exposure to JAK inhibitors during pregnancy or the periconceptional period. Among 25 UC patients (11 maternal and 14 paternal cases), 15 healthy newborns were reported, 2 cases of spontaneous abortion, 2 cases of termination of pregnancy, and no fetal or neonatal death or congenital anomalies.^[165]^ No drug-related adverse pregnancy outcomes were reported in the RA and PsO studies.^[166]^ Data from the baricitinib pregnancy registry (MotherToBaby) indicated that in the limited number of cases with exposure during the first trimester of pregnancy, the pregnancy outcomes of baricitinib (such as spontaneous abortion rate, congenital malformation rate, *etc*.) were similar to those in the general population or other targeted therapies (such as secukinumab, infliximab, and tofacitinib).^[167]^ However, the current data on JAK inhibitor exposure during pregnancy are insufficient to rule out related risks. JAK inhibitors should be avoided during pregnancy. Pharmacokinetic studies during the lactation period indicated that JAK inhibitors were excreted in breast milk.^[168]^ Given the lack of data on the effects of JAK inhibitors on breastfed infants, potential risks cannot be excluded, and avoidance of these agents during breastfeeding is advised. Furthermore, in light of the evidence of teratogenicity in animal studies,^[169]^ women of childbearing potential should take effective contraceptive measures during the use of JAK inhibitors. Female patients should discontinue JAK inhibitors at least 1 month prior to pregnancy planning.

#### (II) Elderly patients

Elderly patients (≥ 65 years) may have decreased liver and kidney function and reduced immunity, leading to an increased risk of infection (*e.g*., TB, pneumonia) and increased risk of cardiovascular events. Adverse events related to JAK inhibitors should be noted, and close monitoring is required.^[170]^

#### (III) Patients with hepatic or renal impairment

Patients with mild to moderate hepatic or renal impairment do not require dosage adjustment when treated with upadacitinib, deucravacitinib, peficitinib, or ivarmacitinib. The specific dose adjustment strategies for JAK inhibitors in the treatment of patients with different liver and kidney function abnormalities are detailed in Table 7.

**Table 7:** Dose adjustment of JAK inhibitors in patients with hepatic or renal impairment

Drug	Metabolic Pathway^a^	Dose adjustment in hepatic impairment	Dose adjustment in renal impairment
Tofacitinib	CYP3A4, CYP2C19	Severe: not recommended.	Moderate to severe: reduce dose to 5 mg once daily.
Baricitinib	CYP3A4	Mild or moderate: no adjustment; severe: not recommended.	CrCL 30–60 mL/min: reduce dose (if 4 mg QD, reduce to 2 mg QD; if 2 mg QD, not recommended); CrCL < 30 mL/min: not recommended.
Upadacitinib	CYP3A4, CYP2D6	Child-Pugh C: not recommended; Child-Pugh A/B: no adjustment for RA, PsA, AD, nr-axSpA, and AS. For UC/CD: induction 30 mg QD, maintenance 15 mg QD.	Severe: RA, PsA, AD, nr-axSpA, and AS: 15 mg QD; UC/CD: induction 30 mg QD, maintenance 15 mg QD; ESRD: not recommended.
Abrocitinib	CYP2C19, CYP2C9, *etc*.	Child-Pugh A/B: no adjustment; Child-Pugh C: not recommended.	Mild: no adjustment; moderate: reduce dose to 50 mg QD; severe: not recommended.
Deucravacitinib	CYP1A2, CYP2B6, *etc*.	Child-Pugh A/B: no adjustment; Child-Pugh C: not recommended.	No adjustment recommended for mild, moderate, or severe impairment, or ESRD patients on dialysis.
Ritlecitinib	GST, CYP3A, *etc*.	Child-Pugh A/B: no adjustment; Child-Pugh C: not recommended.	Not studied in patients with mild or moderate renal impairment or ESRD.
Pevidotinib	SULT2A1, NNMT	Child-Pugh A: no adjustment; Child-Pugh B: initial 50 mg QD (reassess if response is inadequate); Child-Pugh C: not recommended.	No adjustment recommended for mild, moderate, or severe renal impairment.
Ivarmacitinib	CYP3A4, CYP2C8, *etc*.	Child-Pugh A/B: no adjustment; Child-Pugh C: not recommended.	Mild/Moderate: no adjustment; severe or ESRD: not recommended.

a Data are derived from individual product labels. AD, atopic dermatitis; AS, ankylosing spondylitis; CD, Crohn’s disease; CrCl, creatinine clearance; GST, glutathione S-transferase; RA, rheumatoid arthritis; PsA, psoriatic arthritis; nr-axSpA, non-radiographic axial spondyloarthritis; UC, ulcerative colitis.

#### (IV) Patients receiving vaccination

JAK inhibitors increase the risk of infection, so it was recommended to get vaccinated to prevent the reactivation of viruses, such as herpes zoster, and other infectious diseases in patients with IMIDs.^[162]^ A herpes zoster vaccination study in RA patients included RA patients receiving methotrexate therapy; 2 to 3 weeks after vaccination, patients were randomly assigned to the placebo group or the tofacitinib 5 mg treatment group, and the results showed similar vaccine immunogenicity in both groups.^[171]^ Another study included RA patients receiving JAK inhibitor monotherapy, methotrexate monotherapy, or combination therapy, and all patients were vaccinated with the 13-valent pneumococcal conjugate vaccine (PCV13). The results showed that the seropositive response rate in the JAK inhibitor group (95%) was similar to that in the methotrexate group (90%). Combination therapy was the only factor associated with a reduced response to PCV13.^[172]^ Current evidence has not shown that JAK inhibitors have significant adverse effects on vaccine response.^[173,174]^ Therefore, patients receiving inactivated vaccines generally do not require discontinuation of JAK inhibitors.

#### (V) Patients undergoing major surgery

The 2024 China Consensus on JAK inhibitor treatment for rheumatic and immunological diseases states that medication should be discontinued 1 week preoperatively and could be resumed 2 weeks postoperatively, provided there are no issues with wound healing, surgical site, or systemic infection.^[175]^ The German Society for Rheumatology management recommendations indicated that for patients with rheumatic and immunological diseases undergoing major surgery (*e.g*., hip fracture internal fixation surgery, abdominal/pelvic or cardiac operations), JAK inhibitors should be discontinued 3–4 days preoperatively, and the total medication withdrawal period during the perioperative period should not exceed 14 days.^[176]^ The 2022 ACR/American Association of Hip and Knee Surgeons (AAHKS) guideline conditionally recommends that patients with RA, AS, PsA, or JIA should discontinue tofacitinib, baricitinib, and upadacitinib 3 days preoperatively.^[177]^ Clinical studies have provided practical evidence for medication decisions during the perioperative period: a small-sample study showed that among 32 RA patients undergoing orthopedic surgery (49 total surgeries), 31 patients continued JAK inhibitors during the perioperative period, and 18 patients discontinued the medication (average discontinuation period 2.4 days); no surgical site infection was observed during the 90-day postoperative follow-up, and only 1 case of delayed wound healing was reported, with 2 patients experienced disease recurrence 3 and 9 days after discontinuation, respectively.^[178]^ Another retrospective study showed that the disease recurrence rate (35.4%) was higher in the group of RA patients undergoing orthopedic surgery who discontinued JAK inhibitors for ≥ 8 days postoperatively, compared to the group who discontinued for ≤ 7 days (7.1%).^[179]^ A further retrospective study suggested that the overall safety of tofacitinib was controllable in UC patients who received total abdominal colectomy and continued tofacitinib within 4 weeks postoperatively, although the postoperative 90-day VTE incidence was relatively high (13.2%, *n* = 7), indicating that thromboembolic risk should be a key focus during the perioperative use of JAK inhibitors.^[180]^

## Prospection

This consensus provides a systematic combination of the clinical evidence on the application of JAK inhibitors across a spectrum of IMIDs, including rheumatology, dermatology, and gastroenterology. Drawing upon the latest evidence-based medicine and expert clinical experience from China and abroad, this consensus is composed of 18 core recommendations. These statements are intended to offer comprehensive, practical guidance for the appropriate and standardized use of JAK inhibitors in clinical practice. The consensus clearly emphasize that in clinical practice, the principles of “individualized assessment, dynamic monitoring, and risk control” must be followed strictly, with an emphasis on seeking a balance between therapeutic efficacy and safety.

JAK inhibitors hold great promise in the management of IMIDs, with multiple Phase III clinical trials currently underway. Highly selective JAK inhibitors, such as ivarmacitinib, are being actively explored in diseases including nr-axSpA, PsA, vitiligo, and CD. At the same time, with the continuous accumulation of real-world data, the long-term safety of JAK inhibitors will become clear. Future studies should also focus on combination therapy strategies of JAK inhibitors with other targeted drugs and the optimization of dose reduction and discontinuation regimens, in order to achieve more efficient and safe disease management goals. Through ongoing evidence updates and multidisciplinary collaboration, the application of JAK inhibitors is expected to move toward a new stage of standardization and precision.

## Secondary publication declaration

*The Chinese language version of this guideline was published by Chi‑ nese Journal of Internal Medicine in Issue 12 Volume 64, 2025. The current English language version is published after updating and modification with permission from Chinese Journal of Internal Medicine*.
